# Early life stress and LPS interact to modify the mouse cortical transcriptome in the neonatal period

**DOI:** 10.1016/j.bbih.2021.100219

**Published:** 2021-02-13

**Authors:** Eamon Fitzgerald, James P. Boardman, Amanda J. Drake

**Affiliations:** a University/British Heart Foundation Centre for Cardiovascular Science, University of Edinburgh, The Queen’s Medical Research Institute, 47 Little France Crescent, Edinburgh, EH16 4TJ, UK; b MRC Centre for Reproductive Health, University of Edinburgh, The Queen’s Medical Research Institute, 47 Little France Crescent, Edinburgh, EH16 4TJ, UK; c Centre for Clinical Brain Sciences, University of Edinburgh, Chancellor’s Building, 49 Little France Crescent, Edinburgh, EH16 4SB, UK

**Keywords:** Early life stress, Infection, Inflammation, Brain development, Preterm birth, Transcriptomics

## Abstract

**Introduction:**

Preterm birth (PTB) is closely associated with atypical cerebral cortical development and cognitive impairment. Early exposure to extrauterine life often results in atypical environmental and biological experiences that co-occur, including early life stress (ELS) and systemic inflammation. Understanding how these experiences interact to shape cortical development is an essential prerequisite to developing therapeutic interventions that will work in the complex postnatal environment of the preterm infant. Here, we studied the effects of a murine model of infection and ELS on the neonatal cortex transcriptome.

**Methods:**

We used a mouse model of infection (1 ​mg/kg LPS at postnatal day (P)3) +/− ELS (modified maternal separation; MMS on days P4–P6) at timepoints with neurodevelopmental relevance to PTB. We used 4 groups: control, LPS, MMS and LPS ​+ ​MMS. Cortices were dissected at P6 for 3′RNA sequencing.

**Results:**

LPS exposure resulted in reduced weight gain and increased expression of inflammation-associated genes in the brain. More genes were differentially expressed following LPS (15) and MMS (29) than with LPS ​+ ​MMS (8). There was significant overlap between the LPS and MMS datasets, particularly amongst upregulated genes, and when comparing LPS and MMS datasets with LPS ​+ ​MMS. Gene Ontology terms related to the extracellular matrix and cytokine response were enriched following MMS, but not following LPS or LPS ​+ ​MMS. 26 Reactome pathways were enriched in the LPS group, none of which were enriched in the LPS ​+ ​MMS group. Finally, a rank-rank hypergeometric overlap test showed similarities, particularly in upregulated genes, in the LPS and MMS conditions, indicating shared mechanisms.

**Conclusion:**

LPS and MMS interact to modify the cortical transcriptome in the neonatal period. This has important implications for understanding the neural basis of atypical cortical development associated with early exposure to extrauterine life.

## Highlights

• LPS exposure is associated with reduced weight gain in the neonatal period.
• There is an overlap in the transcriptomic response to LPS and early life stress (ELS).
• LPS and ELS modulate the response to each other in the developing male cerebral cortex.

## 1 Introduction

Preterm birth (PTB) associates with an increased risk of atypical neurodevelopment (Johnson and Marlow, 2017). Preterm infants experience a combination of early life stressors (ELS) associating with adverse neurodevelopment including infection, pain and maternal separation (Brummelte et al., 2012; Duerden et al., 2018; Schlapbach et al., 2011). The interactions between these may explain variability in neurodevelopmental outcome, although there are few studies which have addressed this (Fillman et al., 2014).

At birth, murine brain development is at an equivalent stage a human at ~24 weeks gestation, maturing to term equivalence by postnatal day (P)10 (Workman et al., 2013), providing a window when insults can be modelled at timepoints relevant to human PTB. Perinatal infection and ELS have well-characterised effects in the cortex following PTB (Hagberg et al., 2015; Teissier et al., 2020). We hypothesised that commonly experienced insults would interact to affect the developing cortex and we set out to model this using a combination of LPS and ELS.

## 2 Methods

### 2.1 Animal studies

Experiments were performed in accordance with local guidelines and UK Home Office Animals (Scientific Procedures) Act 1986.2 females and 1 male adult C57/BL6J/OLA mice (Harlan, Derby, UK) were housed/cage for breeding. Birthdate was designated P0. On P2, pups were randomly killed, leaving only four males per litter (2 litters/cage). Males were randomised into four groups; control (PBS); LPS; MMS (PBS ​+ ​MMS) and LPS ​+ ​MMS. On P3, PBS (autoclave sterilised) or 1 ​mg/kg LPS (Silva et al., 2019) (Merck Life Science, Dorset, UK; *Escherichia coli* O111:B4) were administered intraperitoneally. MMS was performed (Fitzgerald et al., 2020) between 13:30–15:00 on P4–P6. MMS and LPS ​+ ​MMS pups were placed on a heating pad adjacent to the home-cage, where the control and LPS groups remained. For 1.5 ​h, pups were placed supine whenever they returned to prone. Control and LPS pups remained in the home-cage. For initial characterisation of LPS effects, a group of pups were exposed to PBS or LPS (n ​= ​4/group from 4 litters) and killed by decapitation on P4 before whole brain extraction. For subsequent experiments pups from all 4 groups (n ​= ​10/group from 10 litters) were killed by decapitation immediately after MMS on P6 and cortex dissected without prior perfusion.

### 2.2 Gene expression analysis

RNA was extracted using the Qiagen RNeasy Mini Kit (Qiagen, Manchester, UK). Reverse transcription was performed with the Applied Biosystems RT kit (Thermo Fisher Scientific, Paisley, UK). Primers were designed using the UPL design centre (Supplementary Table 1) and cDNA analysed using a Roche LightCycler 480 (Burgess Hill, UK). Normalisation was done to the housekeeping gene TATA-box binding protein.

From the 10 pups, 3 samples and their littermates were randomly chosen for 3′RNA sequencing at the Wellcome Trust Clinical Research Facility (University of Edinburgh) on the Ion Torrent Platform (Thermo Fisher Scientific, Paisley, UK). Twelve samples were used per chip (average read depth 8,022,074). ~13,453 genes were detected per sample. Raw pH files were converted to flow signals and aligned to the mm10 reference genome using Torrent Suite software (version 5.2.0). Differential expression analysis was done using Limma with voom sample weights (Law et al., 2014) using Degust (Powell). Volcano plots were generated using Galaxy (Afgan et al., 2018) and Heatmaps using ClustVis (Metsalu and Vilo, 2015). Gene Ontology (GO) analysis was performed on genes with fold change >1.5 (Chen et al., 2016; Conesa et al., 2016) using Gprofiler (Raudvere et al., 2019). A Venn diagram for Differentially Expressed Genes (DEGs) was created using InteractiVenn (Heberle et al., 2015). Rank-rank hypergeometric overlap (RRHO) was performed using RRH02 (Cahill et al., 2018) in R version 4.0.2. Geneset enrichment analysis (GSEA) was performed as described (Mootha et al., 2003; Subramanian et al., 2005). Cytoscape 3.8 and EnrichmentMap were used to reconstruct interactions between enriched genesets. Data are available at Gene Expression Omnibus (GSE157184).

### 2.3 Statistical analysis

Differences in candidate gene expression were analysed using independent t-testing. Statistical analyses were performed using IBM SPSS software version 24 or using the specific sequencing software.

## 3 Results

### 3.1 Differential gene expression analysis

LPS exposure was associated with increased expression of inflammation-associated genes in whole brain: *Ionized calcium binding adaptor molecule* 1 (fold change ​= ​2.96, p ​= ​0.013, df ​= ​5), *Interleukin 1α* (fold change ​= ​2.81, p ​= ​0.012, df ​= ​5) and *Tumour Necrosis Factor α* (fold change ​= ​4.17, p ​= ​0.03, df ​= ​5) at the time MMS was commenced. LPS exposure, but not MMS, led to reduced weight gain between P3–P6 (p ​= ​1.07E-05, f(3,39) ​= ​26.136).

**Fig. 1:**
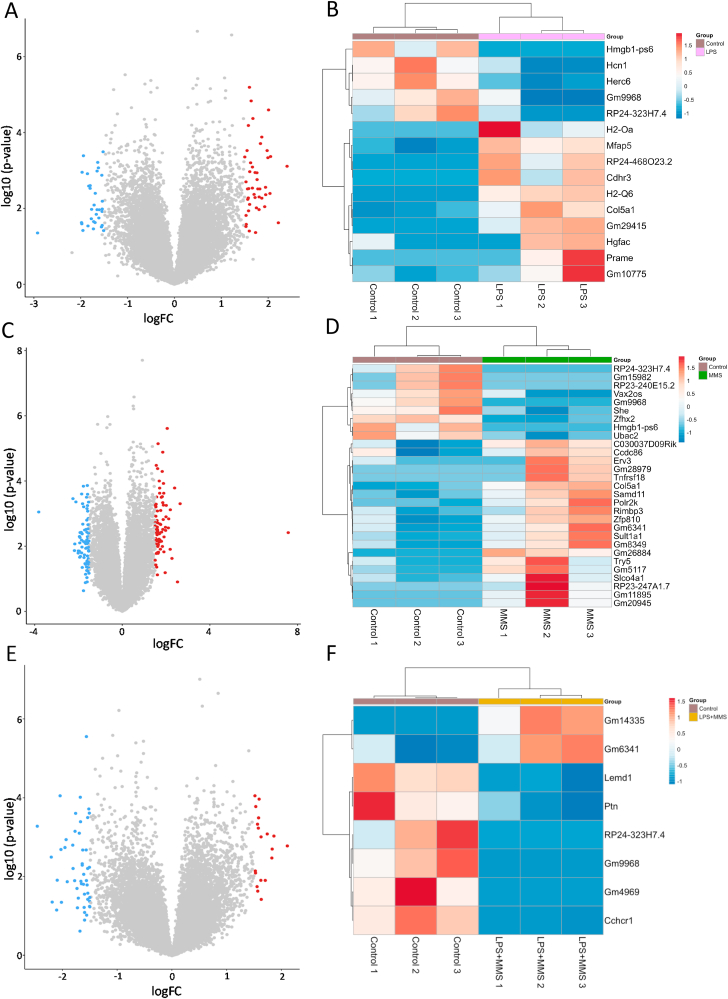
3′ RNA sequencing data from control vs LPS, control vs MMS and control vs LPS ​+ ​MMS comparisons. Volcano plots of differential gene expression analysis (3 samples/group), using Limma with Voom sample weights normalisation, comparing control vs LPS (A), control vs MMS (C) and control vs LPS ​+ ​MMS (E). Log fold change (logFC) on the x-axis, -log(p-value) on the y-axis. Genes with a logFC>1.5 are coloured blue (downregulated) and red (upregulated). (B, D, F) Heatmap of genes with false discovery rate (FDR) ​< ​0.05 from control vs LPS (15 genes), control vs MMS (29 genes) and control vs LPS ​+ ​MMS (8 genes) datasets, respectively. Samples and genes are clustered by Euclidian distance. Blue indicates downregulation; red indicates upregulation of gene expression. (For interpretation of the references to colour in this figure legend, the reader is referred to the Web version of this article.)

**Fig. 2:**
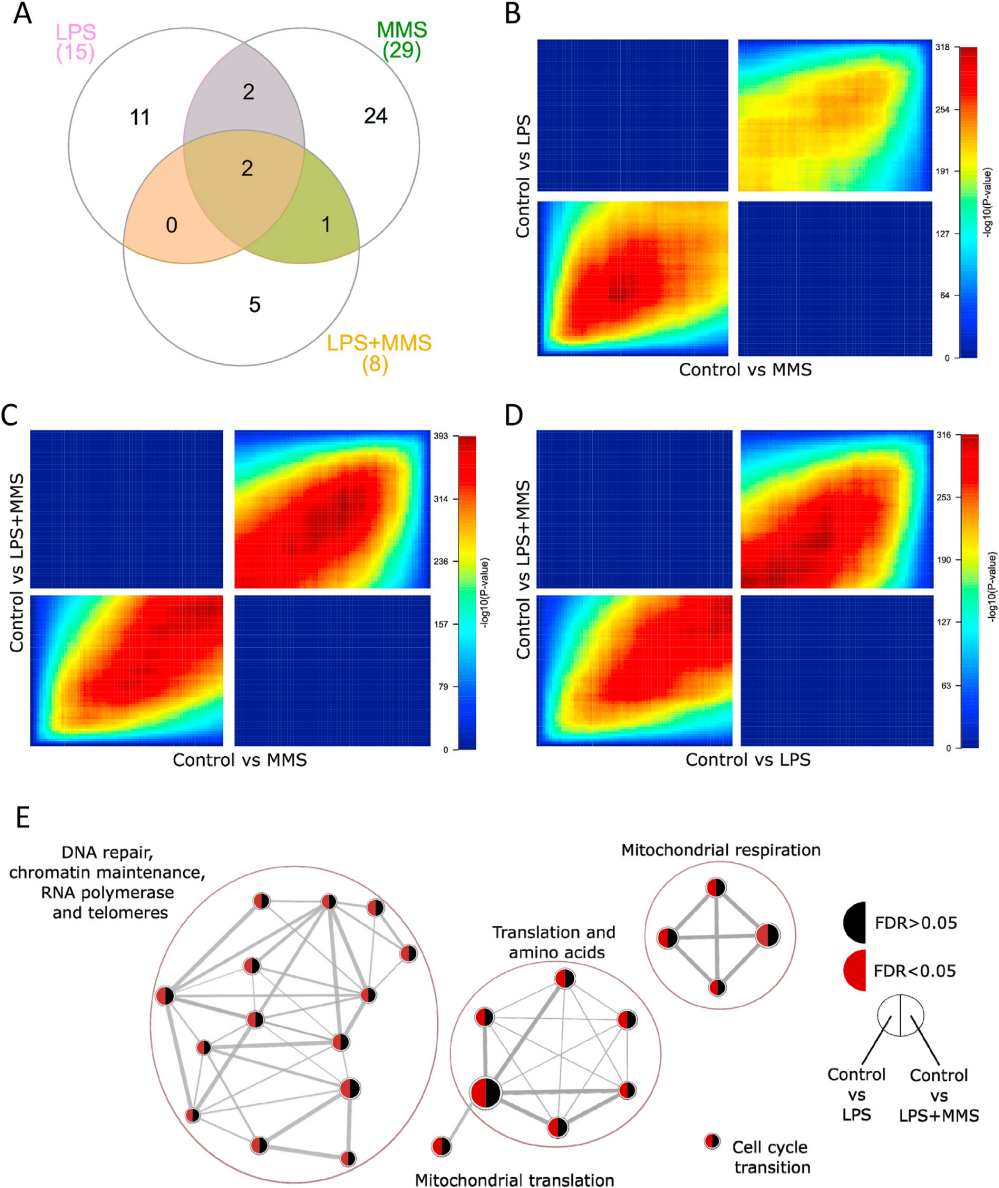
Comparison of Differentially Expressed Genes (DEGs) and ranked gene lists across LPS, MMS and LPS ​+ ​MMS datasets. (A) Venn diagram shows overlap of DEGs (FDR<0.05) between LPS (pink), MMS (green) and LPS ​+ ​MMS (yellow) datasets. (B–D) RRHO analysis of the correlation between (B) LPS and MMS; (C) MMS and LPS ​+ ​MMS and (D) LPS and LPS ​+ ​MMS shows significant subthreshold overlaps. Genes are ranked along the axes: lower left quadrants indicate genes upregulated in both comparisons; upper right quadrants indicate genes downregulated in both comparisons. Each pixel contains an adjusted p-value for the comparison; coloured with respect to the -log10 (p-value) of the overlap. Red and blue indicate the highest and lowest degree of overlap, respectively. (E) Following GSEA 26 genesets were enriched (FDR<0.05) with LPS. There was no enrichment (all terms FDR>0.05) for MMS and LPS ​+ ​MMS datasets. An enrichment map is shown of all significant genesets, each node indicates a unique geneset with the size of the node proportional to the number of genes contained. Edges indicate overlap between nodes with the thickness of the edge corresponding to the degree of overlap. Clusters of nodes are annotated with key words describing the nodes contained within them. Each node contains an indication of statistical significance for the LPS (left) and LPS ​+ ​MMS (right) datasets. Red indicates FDR<0.05; black indicates FDR>0.05.3 samples/group were sequenced. (For interpretation of the references to colour in this figure legend, the reader is referred to the Web version of this article.)

Compared with controls at P6: LPS exposure associated with differential expression of 15 genes (FDR<0.05) (Fig. 1A and B) and MMS exposure with differential expression of 29 genes (Fig. 1C and D); 8 genes were differentially expressed with combined LPS and MMS (Fig. 1E and F). 4/15 LPS DEGs were also differentially expressed with MMS and 2/15 overlapped with LPS ​+ ​MMS (Fig. 2A). 3/29 MMS DEGs were also differentially expressed with LPS ​+ ​MMS. *RP24-323H7.4* and *GM9968* were differentially expressed in all groups. Analysis of dataset overlap using RRHO revealed significant overlap between LPS and MMS datasets (Fig. 2B) and also when comparing LPS alone and MMS alone with LPS ​+ ​MMS (Fig. 2C and D).

### 3.2 GO analysis shows modulation of the MMS response by LPS

GO analysis (Supplemental Figure 1) identified one enriched term (adjusted p-value <0.05) with LPS: ‘Transition metal ion binding’, in keeping with literature showing sequestration of transition metals following bacterial infection (Hood and Skaar, 2012). Terms enriched with MMS included ‘response to cytokine’ and terms related to the extra-cellular matrix (ECM). ‘Intrinsic component of the plasma membrane’ and ‘Extracellular region’ were enriched with LPS ​+ ​MMS.

### 3.3 Functional pathway analysis suggests that MMS modulates the LPS response

GSEA analysis of enrichment (FDR<0.05) within the Reactome database identified 26 genesets enriched with LPS (Fig. 2E) including clusters related to DNA repair, chromatin maintenance, RNA polymerase/telomeres; mitochondrial respiration; translation/amino acids; mitochondrial translation; and cell cycle transition (Supplementary Table 2). No enrichment was seen in MMS or LPS ​+ ​MMS datasets.

## 4 Discussion

Using an unbiased sequencing-based approach, we describe a reciprocal modulation of LPS and ELS responses, with LPS ​+ ​MMS resulting in fewer DEGs and enriched terms on GO and GSEA analysis than either alone. The significant overlap between LPS and MMS suggests shared mechanisms.

LPS associated with altered expression of *HCN1* (*Hyperpolarization Activated Cyclic Nucleotide Gated Potassium Channel 1*); previous studies have shown LPS-induced neuroinflammation associates with reduced HCN1 and altered HCN1 function leads to abnormalities implicated in hyperexcitability and cognitive deficits (Frigerio et al., 2018; Marini et al., 2018). LPS also induced expression of *Microfibrillar-associated protein 5* and *Col5a1,* both components of the ECM (Gibson et al., 1998; Takahara et al., 1991). The ECM is important in cell differentiation/migration, axonal outgrowth and synaptic connectivity (Barros et al., 2011; Long et al., 2018; Nguyen et al., 2020) and is implicated in psychiatric disorders and age-related cognitive deficits (Pantazopoulos and Berretta, 2016). Analysis using the Reactome database suggests LPS leads to dysregulation of cell processes with roles including DNA repair (Kidane et al., 2014), transcription (Hagberg et al., 2015), translation (Hagberg et al., 2015), mitochondrial respiration (Eisenreich et al., 2019) and cell cycle (Oswald et al., 2005).

Among the DEGs associated with MMS was Zfhx2 (Zinc Finger Homeobox 2), which plays a role in neuronal differentiation (Komine et al., 2012) and is expressed in sensory neurons (Habib et al., 2017); abnormalities in Zfhx2 associate with behavioural abnormalities including hyperactivity and anxiety (Komine et al., 2012). This is of interest as we identified stress-induced hyperactivity following MMS (Fitzgerald et al., 2020). We also observed enrichment of the GO term ‘response to cytokine’ with MMS. Further studies are required, including protein analysis, in order to determine the directionality of this response, for example whether this represents a decrease in proinflammatory or an increase in anti-inflammatory cytokine signalling.

Although we identified fewer DEGs following LPS ​+ ​MMS than with either alone, RRHO analysis indicated significant sub-threshold overlaps between groups (Fig. 2B–D), implying shared mechanisms. This is supported by studies implicating inflammatory mechanisms in stress responses in adult brains (Koo et al., 2010). Functional pathway analysis indicates modulation of the LPS response by MMS. In the rat hippocampus, stress potentiates gene expression changes to LPS (Bekhbat et al., 2019) and Interferon (IFN) β administration during pregnancy potentiates the effects of ELS in rodents (Ben-Yehuda et al., 2020). Overall, our data show that combined LPS ​+ ​MMS results in less transcriptional perturbation than either alone and suggests that prior LPS produces an environment that is less permissive to MMS and/or that under these conditions MMS has anti-inflammatory effects. Further work is needed to establish the nature and directionality of these interactions which have implications for the development of therapeutics, indeed anti-inflammatory drugs protect the neonatal rodent brain from the effects of stress (Bronson and Bale, 2014).

There were some study limitations. Resolution of LPS-induced inflammation begins after 24 ​h (Erickson and Banks, 2011). Nevertheless, in our study, analysis of inflammatory gene expression suggests a pro-inflammatory environment was present in whole brain at the onset of MMS. Additionally, ongoing weight gain was affected, suggesting that LPS had a physiological effect which persisted through MMS. The interaction paradigm could also be explained by LPS-induced ‘pre-conditioning’. Although male-specific effects have been described following neonatal infection (Imahara et al., 2005; O’Driscoll et al., 2018; O’Driscoll et al., 2017), these experiments should be repeated in females. Perfusion was not performed, future studies are necessary to evaluate any potential effects of inflammatory changes in blood cells which may influence gene expression in brain tissue. Further work is also needed to evaluate adult behaviour and whether effects are limited to specific brain subregions.

In conclusion, we demonstrate that LPS and ELS reciprocally modulate each other in the perinatal cortex. This has implications for understanding the pathogenesis of atypical neurodevelopment associated with PTB. Better understanding of underlying mechanisms will be beneficial for the development of therapeutics and improved clinical care.

## Declarations of competing interest

None.
