# Supplementary material for: Early life stress and LPS interact to modify the mouse cortical transcriptome in the neonatal period
Source: Brain Behav Immun Health. 2021 Feb 13;13:100219. doi: 10.1016/j.bbih.2021.100219 (PMC8474587; doi:10.1016/j.bbih.2021.100219)
Supplement: Multimedia component 1 [file mmc1.pptx]

## Slide 1
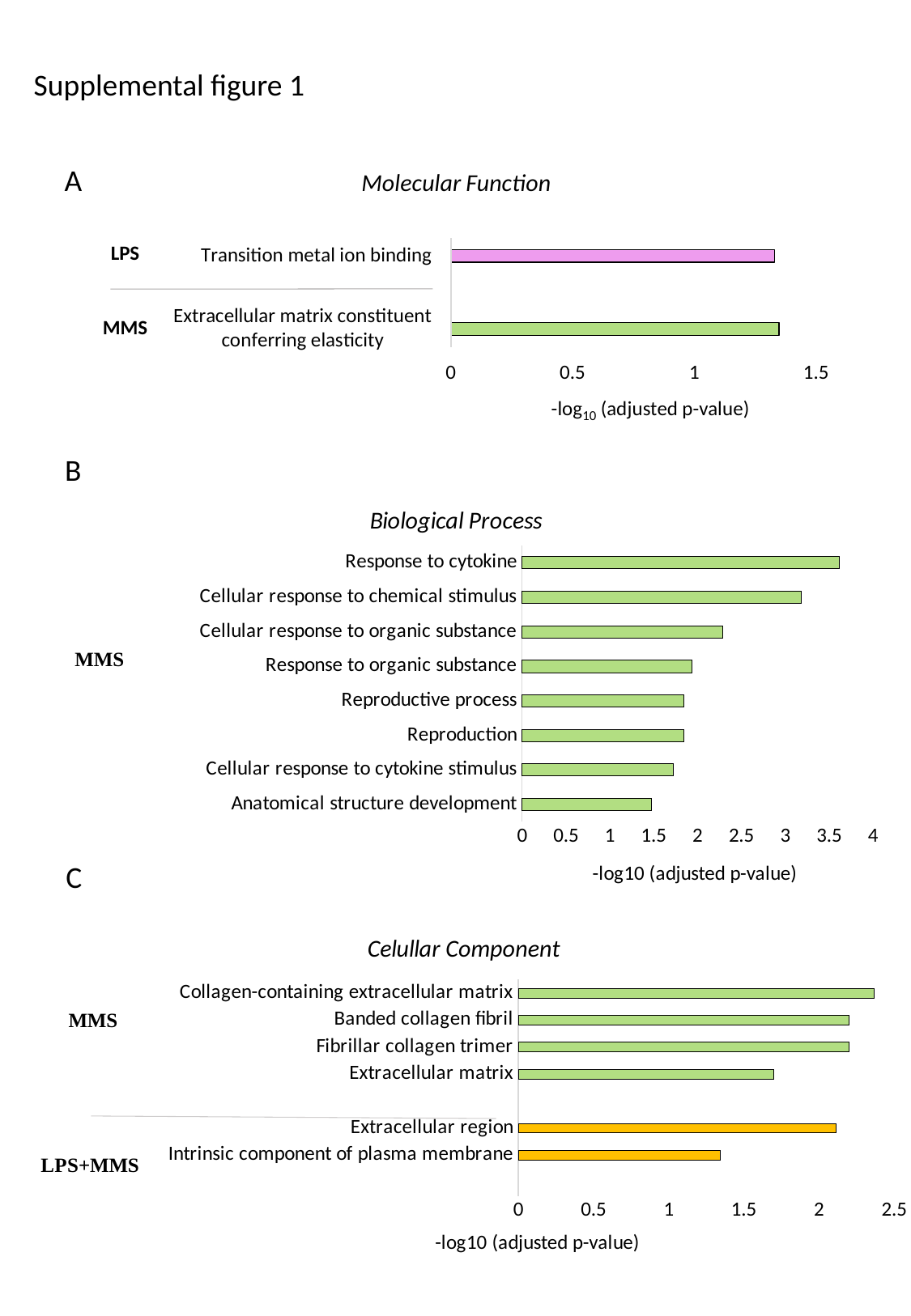

Supplemental figure 1
A
B
### Chart: Biological Process
| Category | |
|---|---|
| Anatomical structure development | 1.47354740284615 |
| Cellular response to cytokine stimulus | 1.72292473319884 |
| Reproduction | 1.84120192828684 |
| Reproductive process | 1.84819455375903 |
| Response to organic substance | 1.93796929614641 |
| Cellular response to organic substance | 2.28646459935024 |
| Cellular response to chemical stimulus | 3.18224044891785 |
| Response to cytokine | 3.61320748732883 |C
### Chart: Celullar Component
| Category | |
|---|---|
| | None |
| Intrinsic component of plasma membrane | 1.34438147003914 |
| Extracellular region | 2.11474772991546 |
| | None |
| Extracellular matrix | 1.69744172581898 |
| Fibrillar collagen trimer | 2.20308790520934 |
| Banded collagen fibril | 2.20308790520934 |
| Collagen-containing extracellular matrix | 2.36610125467163 |
