# Supplementary figures and images for: Early life stress and LPS interact to modify the mouse cortical transcriptome in the neonatal period
Source: Brain Behav Immun Health. 2021 Feb 13;13:100219. doi: 10.1016/j.bbih.2021.100219 (PMC8474587; doi:10.1016/j.bbih.2021.100219)

## Slide 1
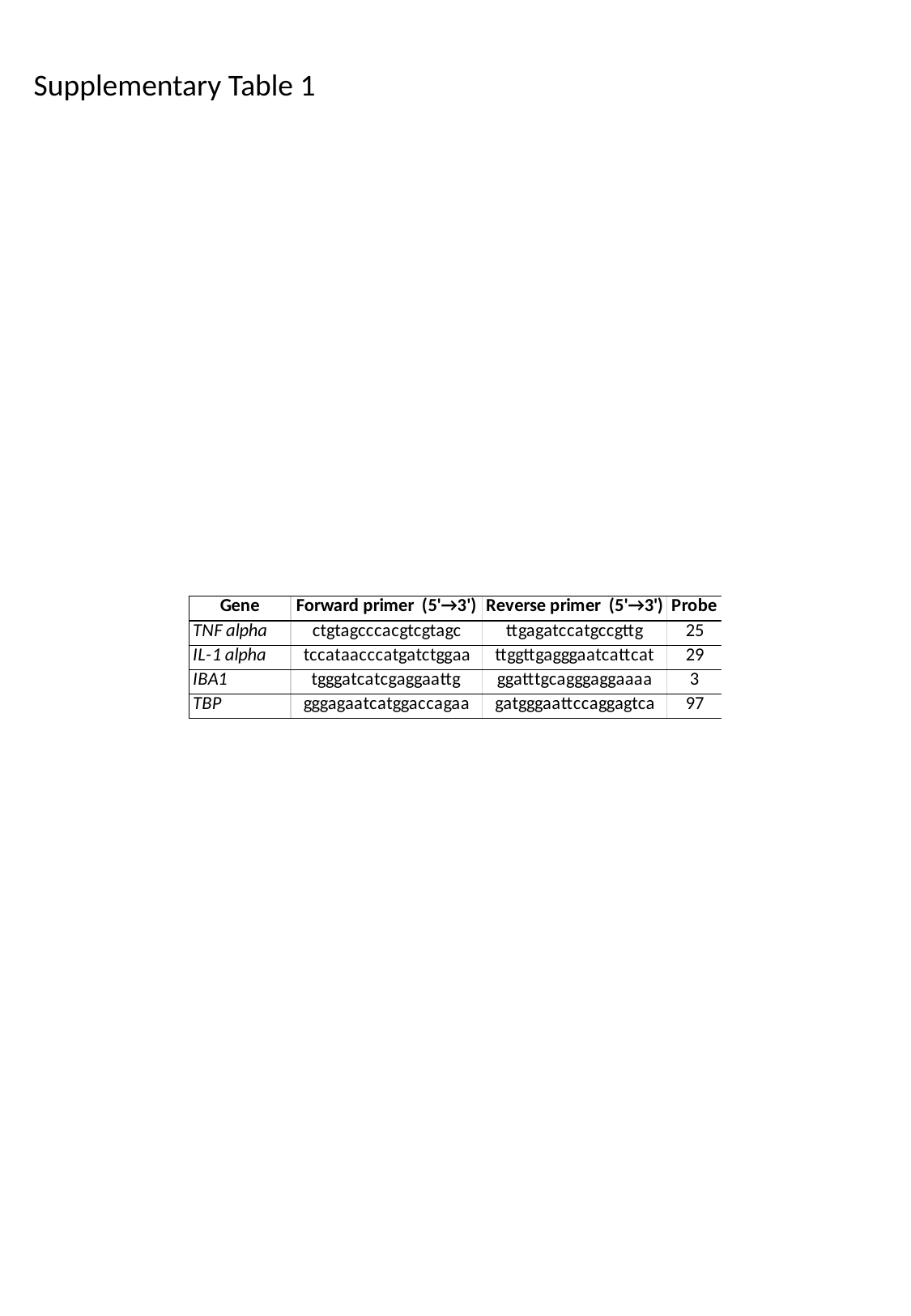

Supplementary Table 1

Supplement: Multimedia component 2 [file mmc2.pptx]

## Slide 1
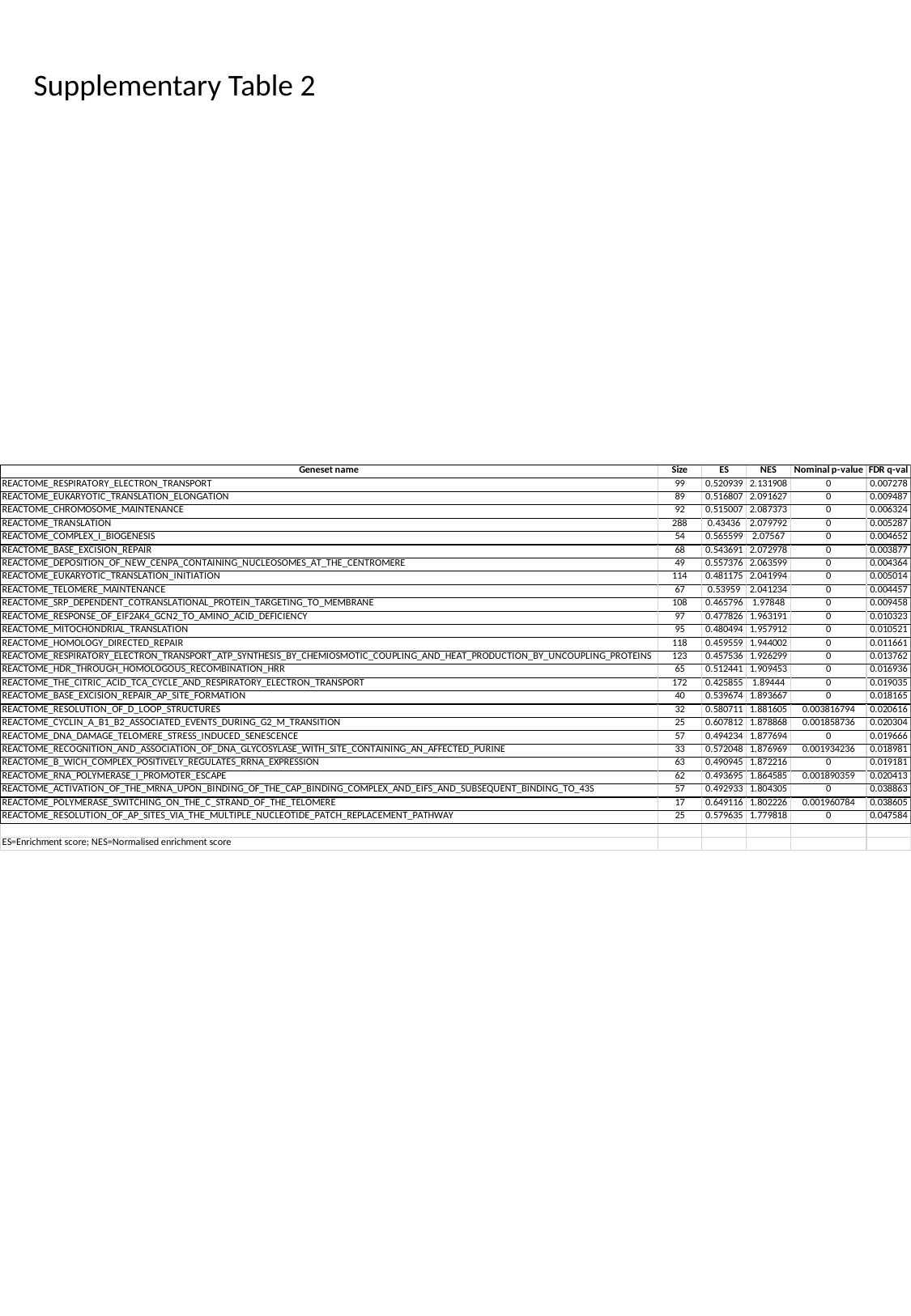

Supplementary Table 2

Supplement: Multimedia component 3 [file mmc3.pptx]
